# Deep learning for stress oriented human activity recognition

**DOI:** 10.3389/fdgth.2026.1729804

**Published:** 2026-05-18

**Authors:** Muhammad Hamza, Nasir Uddin, Gulnaz Anjum, Mohammad Anas, Uzair Gabol, Nida Saddaf Khan

**Affiliations:** ^1^ Department of Computer Science, National University of Computer and Emerging Sciences, Karachi, Pakistan; ^2^ Department of Psychology, University of Oslo, Oslo, Norway; ^3^ University of Texas Health Science Center, Houston, TX, United States

**Keywords:** human activity recognition (HAR), long short-term memory (LSTM), mental health, recurrent neural networks (RNNs), stress detection, transformers

## Abstract

**Introduction:**

Human Activity Recognition (HAR) using sensor-generated time-series data has gained significant attention for assessing mental and physical states to address various behavioral disorders. This study utilizes benchmark datasets of stress-related activities to improve prediction performance.

**Methods:**

To achieve this, we employ multiple deep learning architectures, including Recurrent Neural Networks (RNNs), Long Short-Term Memory (LSTM) networks, and Transformer models, for feature extraction and classification. Comprehensive experiments are conducted to evaluate model performance, with particular focus on the impact of window size and overlap ratio on classification accuracy.

**Results:**

The experimental results demonstrate that Transformer models outperform LSTM and RNN models, achieving classification accuracies of 97.83%, 97.36%, and 92.4% on the test dataset, respectively. Furthermore, the proposed approach shows a significant improvement over the deep neural network reported in the original Stressense dataset study.

**Discussion:**

These findings highlight the effectiveness of Transformer-based architectures for HAR tasks involving stress detection. The improvement in classification performance suggests strong potential for advancing seamless mental health monitoring using non-intrusive wearable devices.

## 1 Introduction

Human Activity Recognition (HAR) is a transformative field which involves learning complex patterns and learning temporal dependencies, enabling the classification of activities like walking, writing, or typing based on sensor data [1, 2]. With the widespread use of mobile devices and smartwatches, time-series sensor data is now more accessible than ever, making HAR a cornerstone of applications in fitness tracking, healthcare, and smart environments. Sensors such as accelerometers, magnetometers, and gyroscopes are commonly employed, capturing multi-dimensional data across *x*, *y*, and *z* coordinates [3–5]. This rich, high-resolution data is then fed into advanced machine learning or deep learning models to accurately identify the activity being performed.

Stress related diseases are becoming more and more common in the domain of medical Health. In the recent work [6] held in 2020 in USA shows that 3 out of 4 people (75%) suffers from stress in their daily lives while it was 66% in 2019 i.e that more people are facing stress in their daily lives with the passage of time. Stress often manifests in an individual’s daily behavior, making it possible to identify through observable actions. Self-Regulatory Coping Behaviors (SRCB), such as smoking or overeating, can provide valuable insights into an individual’s stress levels [7]. Additionally, people experiencing stress frequently engage in Body Focused Repetitive Behaviors (BFRBs), such as touching their face or biting their nails [8].

Human Activity Recognition (HAR) offers a promising approach to monitoring and analyzing behaviors associated with stress. By leveraging data from sensors like accelerometers, gyroscopes, and magnetometers embedded in wearable devices, HAR can detect subtle patterns in movements and actions that may indicate stress-related behaviors [4]. For instance, repetitive hand movements, changes in posture, or increased activity in specific body parts can be tracked and analyzed. Recently, researchers have prepared a dataset specifically for the stress related behaviors based on time series data that can be used to perform Human Activity Recognition [9].

In this study, we perform comprehensive experiments using state-of-the-art deep learning architectures, including Recurrent Neural Networks (RNNs), Long Short-Term Memory (LSTM) networks, and Transformers, to tackle stress-oriented Human Activity Recognition (HAR). We examine a range of window sizes from 16 to 512 instances, with overlaps varying between 50% and 80%, to assess how these parameters influence model performance. This systematic variation allows for a thorough investigation of how different windowing strategies influence temporal feature extraction and model efficiency. Our experiments aim to understand how changes in window size and overlap affect the classification accuracy and efficiency of stress-related activities, providing valuable insights into the optimal configuration for stress detection. The best-performing results were selected for each model at different window sizes, ensuring a fair comparison.

Our work is structured around the following main modules for HAR on time-series data:

- **Feature Extraction**: In this module, relevant features are extracted from the time-series data using deep learning models. These features serve as the foundation for the subsequent classification process.
- **Attention Mechanism**: This module is specific to the Transformer architecture and enhances the model’s focus on the most informative parts of the data. By assigning more weight to crucial information, it helps improve the accuracy of activity classification.
- **Classification**: The classification module is responsible for categorizing the activity based on the features extracted in the previous steps. It uses these features to assign the correct label to the input data, enabling accurate stress-oriented activity recognition.

## 2 Related work

Human Activity Recognition (HAR) has emerged as an important paradigm for analyzing human behavioral patterns through sensor-driven data analysis. Traditional HAR research primarily focuses on recognizing physical activities such as walking, sitting, or typing; however, recent studies have explored its applicability for assessing mental and physiological states, including stress. Stress often manifests through subtle behavioral and motor variations, such as changes in movement intensity and gesture dynamics, making HAR a suitable framework for indirectly analyzing stress-related behavioral responses. In controlled experimental settings, where activities are recorded in isolation, HAR enables systematic observation of activity-specific motion patterns while minimizing environmental and contextual noise. Such controlled acquisition allows researchers to better examine how stress influences movement characteristics within individual activities, providing a structured foundation for modeling stress-related behavioral variations using wearable sensor data.

Research conducted by Daniel et al. (2023) [10] explored various traditional machine learning algorithms using data collected from multiple sensors. The dataset included activities such as walking, driving, and brushing teeth. Experiments were performed with algorithms including Support Vector Machines (SVM), Decision Trees (DT), Naive Bayes (NB), K-Nearest Neighbors (KNN), and Random Forest (RF). Among these, Random Forest achieved the highest accuracy of 92.97%. Similarly, in 2016, researchers [2] collected data from various sensors embedded in mobile phones, including accelerometers, gyroscopes, and magnetometers. They evaluated the impact of different window sizes on activity recognition, with the time intervals spanning from 2 to 30 s. The dataset consisted of 13 distinct activities, and to assess the performance of the model, they employed several traditional machine learning algorithms, including Naive Bayes (NB), K-Nearest Neighbors (KNN), and Decision Trees (DT), aiming to identify the most effective approach for recognizing these activities. These studies collectively demonstrate that traditional machine learning approaches can effectively recognize well-defined physical activities; however, their performance often depends heavily on handcrafted features.

In addition to the earlier mentioned applications, researchers have also applied these techniques to classify various movements in sports, such as dab, drive, short serve, smash in badminton [5]. In the study by Tim et al. [5], Convolutional Neural Networks (CNNs) were utilized to accurately categorize different hand movements, including actions such as the smash, dab, and short serve, demonstrating the potential of CNNs in classifying dynamic movements. A more extensive approach is explored in [11], where a combination of Convolutional Neural Networks (CNNs), Bi-directional Gated Recurrent Unit (Bi-GRU), and an attention mechanism was employed to enhance the performance of Human Activity Recognition (HAR) on the UCI-DSA dataset [12]. This model, known as the CNN-BiGRU-CBAM, achieved impressive results, attaining an accuracy and F1-score of 99.1%, underscoring the efficacy of integrating these advanced techniques for recognizing complex human activities.

Additonally, Temporal Convolutional Neural Networks (TCNs) have been tested on several datasets, including WISDM-v1 [13], Active Miles, and Skoda, with different configurations of nodes and filters, consistently achieving over 90% accuracy across all datasets [11]. HAR has also found applications in the medical field, such as in studies on Parkinson’s disease, where the performance of models was evaluated using a weighted F1-score. The Bi-directional LSTM model outperformed others, achieving a weighted F1-score of 0.927 [14]. Furthermore, in 2024, stress-related activities were collected from 30-40 participants using wrist-worn sensors [9]. Extensive experimentation with this dataset involved the application of both Deep Neural Networks (DNNs) and traditional machine learning algorithms [4], where DNNs demonstrated superior performance, achieving a test accuracy of 93.5%.

Mobile phones were used in order to perform Human Activity Recognition for daily life activities including walking, standing, sitting, with Deep Neural Networks (DNN) [1]. In 2015, Vepakomma et al. proposed a framework entitled Wristocracy to categorize 22 different complex activities by using multiple modalities such as environmental information and location context using Bluetooth [15]. Similarly, Abreu et al. in [3] used Hidden Markov Models (HMM) to predict 10 complex activities after gathering data from accelerometer, gyroscope, magnetometer, and microphone.

Research conducted conducted by Casey et al. [16] proposed an Artificial Neural Network (ANN) in order to distinguish smoking from different activities. Moreover, they also performed an analysis of the coordinates of the gyroscopes and concluded that the x-dimension was more useful when predicting the smoking activity. Recent work in [17] performed feature extraction with a combination of CNNs and Bi-directional Gated Recurrent Units (Bi-GRU), which were then given as input to a Single Feed Forward Neural Network for HAR. Research in [18] discovers a new method for gathering data using pedals rather than mobile sensors and wristwatches. The Differential Temporal LSTMs (DF-LSTMs) were used to classify activities including sitting, walking up and down the stairs, and others.

Synthetic data was created using Generative Adversarial Networks (GANs) to train deep learning models, including LSTMs and GRUs [19]. LSTMs were found to be more efficient and accurate when evaluated on the WISM and MHealth datasets. In 2023, Talaat et al. gathered data using Internet of Things (IoT) devices and performed preprocessing to filter out noise [20]. They compared various traditional machine learning algorithms, where Random Forest outperformed the others. Furthermore, Bi-directional LSTMs were employed to develop a monitoring model for stress-related activities in [21].

Transformers have emerged as a groundbreaking architecture with widespread applications, starting with their success in Natural Language Processing (NLP) [22] and later gaining prominence in fields like computer vision as Vision Transformers [23] and sensor-based data analysis. In 2022, researchers introduced two lightweight Transformer-based models, HART and Mobile HART [24], which showcased superior efficiency across five datasets [24]. However, a 2024 study [25] critically evaluated Transformers for Human Activity Recognition (HAR) and concluded that, while powerful, they are highly memory-intensive and not always the most efficient choice.

While advanced architectures such as Transformers have shown strong performance, the effectiveness of any HAR system is also strongly influenced by the characteristics of the sensing modality. In particular, wrist-based wearable sensors, although practical and widely used, introduce several challenges for activity recognition. Wrist-mounted devices are very sensitive to small and unrelated hand movements, which often produce noisy signals and similar patterns across different activities. Actions such as writing, typing, or simple hand gestures can generate nearly identical motion signals, making it harder for models to clearly separate activity classes during training. This difficulty becomes even greater in stress detection, where many activities involve limited physical movement or repetitive motions.

## 3 Dataset

### 3.1 Dataset collection

In this study, human activity recognition (HAR) experiments were conducted using the dataset introduced in [9]. Consistent with our previous work, the dataset contains the same five stress-related activities, namely smoking, eating, face touching, nail-biting, and remaining still. The data were collected from 40 healthy right-handed participants (20 male and 20 female) aged between 20 and 25 years using a custom Android application, with sensor signals recorded at a sampling rate of 50 Hz, which adequately captures the frequency range of typical human movements. Participants performed a set of structured stress-related and baseline activities within a controlled environment. Activity recordings were manually initiated and terminated through the application, and each activity was captured independently to ensure clear annotation. Each activity was recorded in isolation rather than embedded within continuous daily-life activity sequences, allowing precise segmentation and labeling. Labels were assigned automatically based on the activity selected during recording, resulting in consistently labeled segments across participants. The dataset contains approximately 500,000 time-series instances and comprises 12 columns: a user identifier, timestamp, activity label, and nine sensor channels derived from accelerometer, gyroscope, and magnetometer measurements, each represented along three spatial axes (*x*, *y*, and *z*). While the distribution of samples across activities is nearly balanced as shown in Table 1, the number of recordings per participant varies because not all individuals performed every activity. Data collection followed ethical guidelines, including informed consent procedures, and participants were free to discontinue participation at any time.

**Table 1:** Distribution of instances across activity classes.

Activity	Number of instances
Staying still	120,063
Nail biting	111,189
Smoking	94,586
Eating	85,188
Face touching	84,420

### 3.2 About dataset

Smoking was defined as a sequence of hand-to-mouth movements in which participants raised a cigarette, inhaled, and returned the hand to a resting position, with recording continuing until a full cigarette was consumed. Eating was captured in a controlled setting using chocolate consumption to minimize variability caused by cultural dining practices, utensil usage, and individual eating habits, and recording continued until the food item was finished. Nail-biting, commonly associated with stress or nervous behavior, was performed by habitual participants in both seated and standing positions for a duration of three minutes, emphasizing wrist movements occurring near the face. Face-touching involved natural interactions with different facial regions, including areas such as the eyes, nose, mouth, hair, and chin, and participants were instructed to behave normally while recordings were conducted for three minutes in both sitting and standing postures. Remaining still served as a baseline condition in which participants stayed largely inactive while allowing natural minor movements such as posture adjustments or subtle fidgeting, with recordings also lasting three minutes across both postures.

The selection of these activities was motivated by their strong behavioral and motion similarity, which makes them inherently challenging to distinguish using wrist-worn sensor data. Many of the chosen actions involve comparable hand-to-face or hand-to-mouth movements, resulting in overlapping motion patterns and highly correlated sensor signals. Rather than selecting activities that are easily separable, the study intentionally focuses on subtle and closely related behaviors to evaluate the model’s ability to learn fine-grained temporal dynamics. This design encourages the models to capture sequential dependencies and nuanced motion characteristics instead of relying on simple positional or amplitude-based differences. By emphasizing difficult-to-separate activities, the objective was to develop a more robust recognition system capable of extracting meaningful temporal features, thereby better reflecting realistic challenges encountered in stress-related human activity recognition scenarios.

### 3.3 Preprocessing

To ensure better training performance and faster convergence, we standardize the data. First, we select the instances corresponding to a specific user ID and activity. Once the relevant data is extracted, we apply standardization to each feature of the time-series data. The standardization process transforms the data to have a mean of zero and a variance of one, which helps in reducing the impact of varying scales across features and accelerates the learning process.

**Figure 1:**
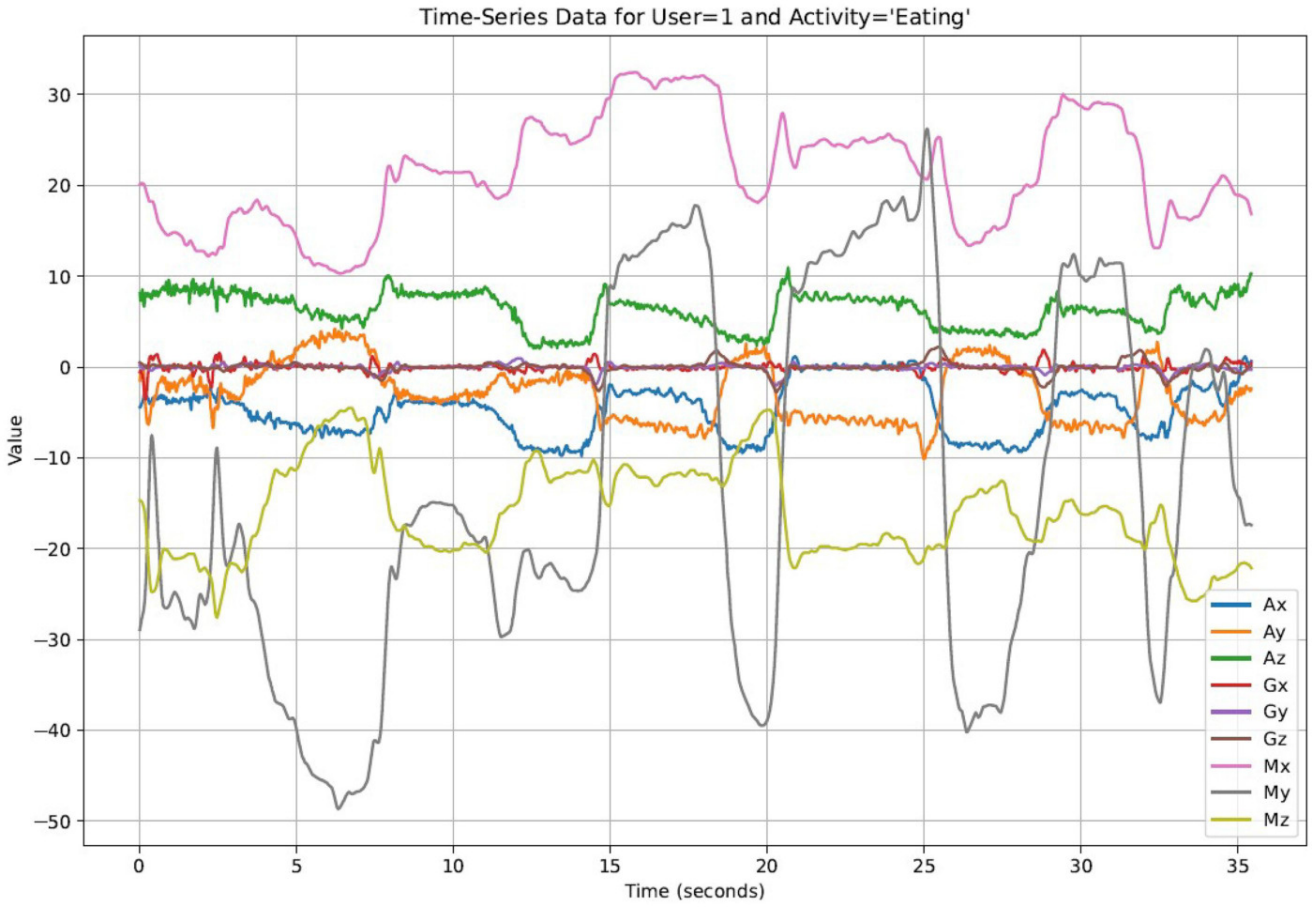
Unstandardized data.

**Figure 2:**
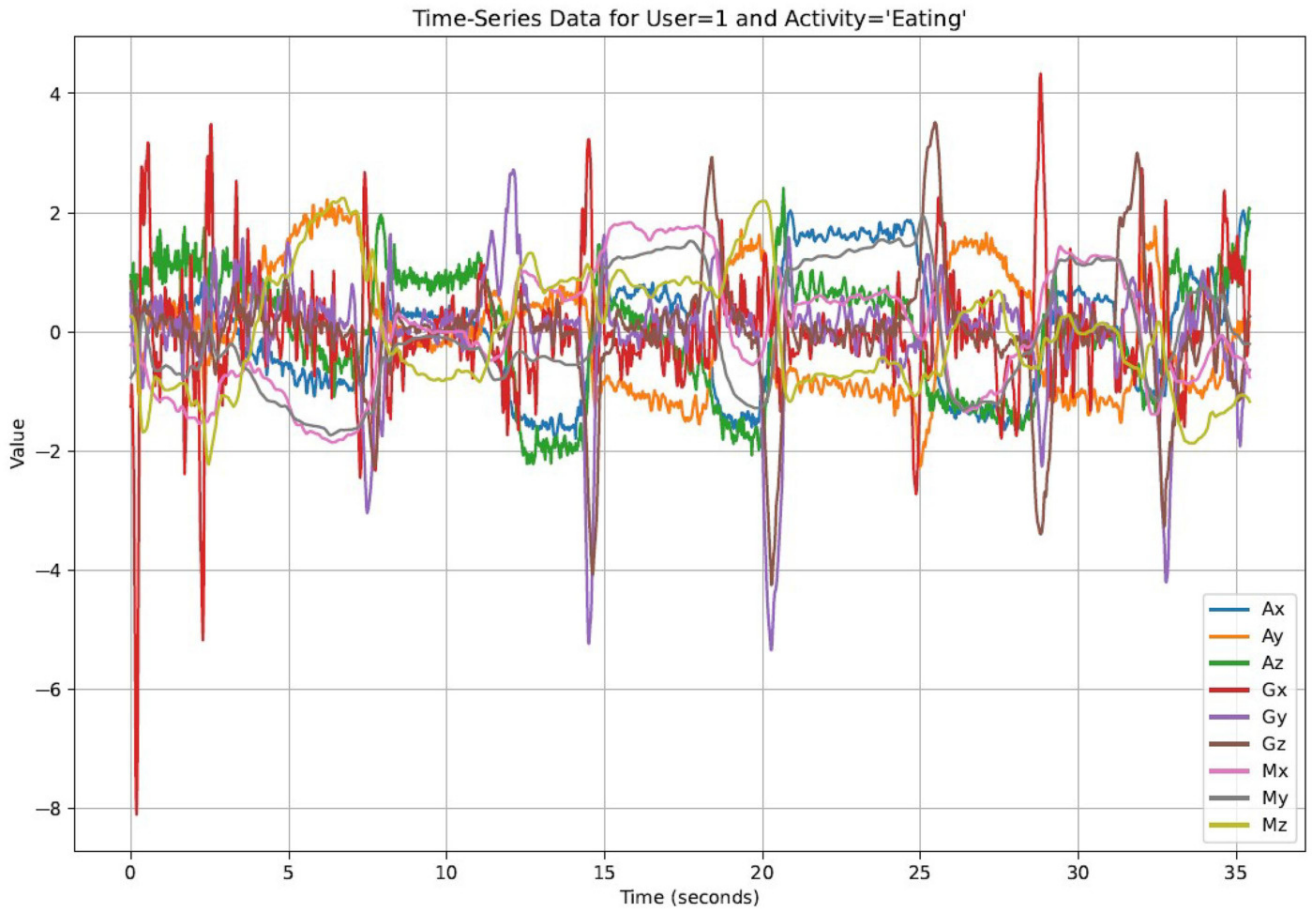
Standardized data.

The standardization formula is given by:

$$z=\frac{x-\mu }{\sigma }$$ (1)

where z is the standardized value, x is the original value, μ is the mean, and σ is the standard deviation of the data for each specific feature. Equation 1 illustrates how each coordinate of the time-series data for a specific stress-related activity is standardized to make the dataset more suitable training deep learning model. Figure 1 illustrates the appearance of the raw data, while Figure 2 depicts the time series data after standardization.

**Figure 3:**
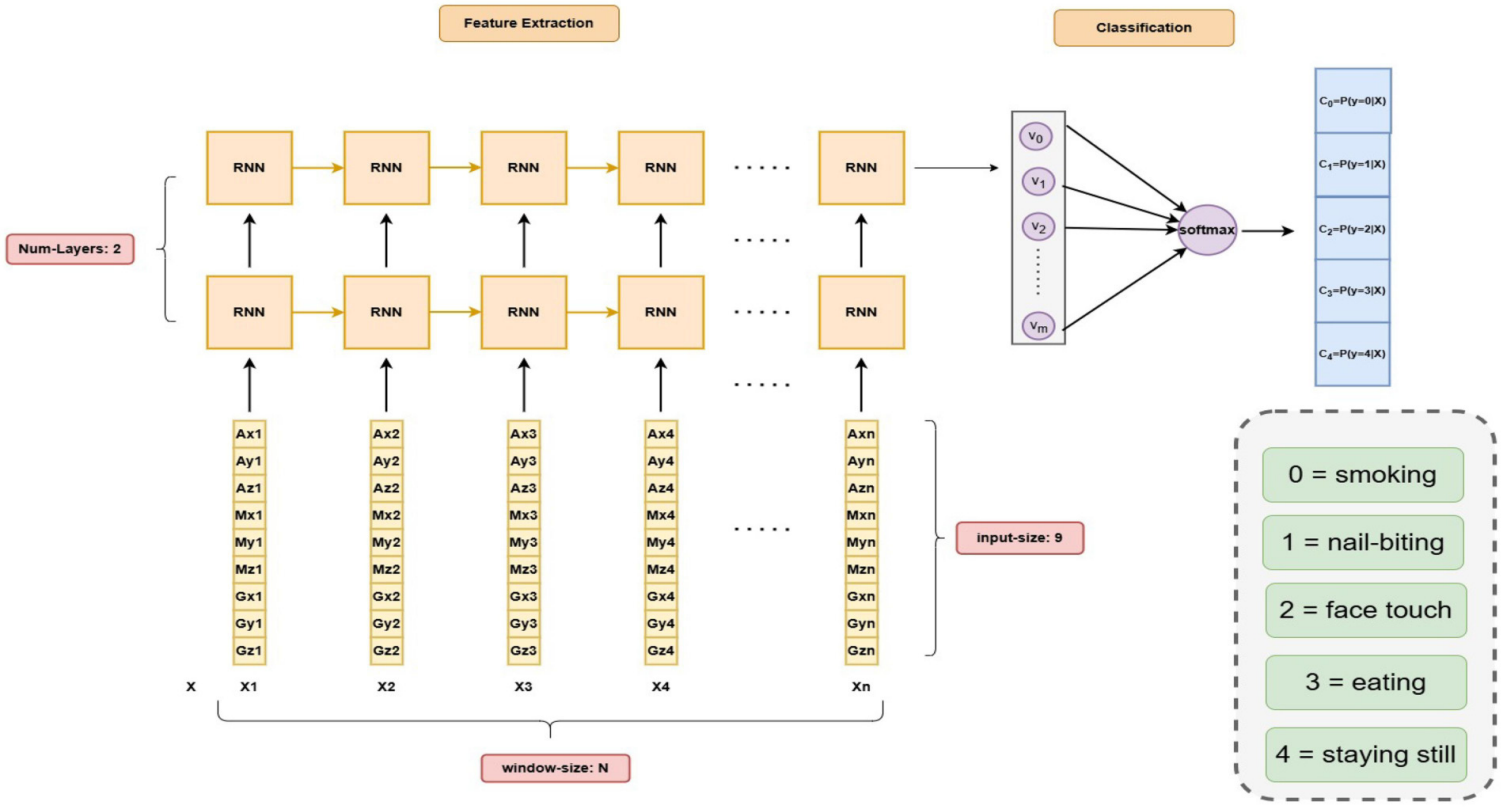
Human activity recognition using deep RNN/LSTMs, adapted to the sensor dataset from Qadir et al. [9].

### 3.4 Dataset preparation

After normalizing the data, we prepared the dataset by segmenting it into windows based on activity and user ID. These windows vary in size, ranging from 16 to 512 instances. The use of window sizes, rather than single instances, is crucial as it enables the deep learning model to capture temporal dependencies and sequential patterns within the data, which are important for recognizing complex activities. Additionally, for each window size, we created four different versions of the dataset with overlapping rates of 50%, 60%, 70%, and 80%. This approach allows us to analyze the impact of different window sizes and overlap percentages on the performance of the model.

## 4 Methodology

Human Activity Recognition is based on time series sensor data, in which individual readings are temporally dependent and only meaningful when considered in sequence. Accurate activity classification therefore requires modeling these temporal dependencies, as single observations do not provide sufficient context to determine the performed activity. Previous work has applied deep neural networks to this task; however, such approaches may struggle to preserve temporal order and long-term dependencies across sequences [4]. Motivated by the inherent sequential nature of human movements, we employ sequence-based models, including Recurrent Neural Networks (RNNs), Long Short-Term Memory networks (LSTMs), and Transformers, to capture contiguous patterns in the data. Since our objective is activity classification rather than sequence generation, we adopt encoder-only architectures, enabling the extraction of rich temporal representations that effectively leverage the sequential structure of human movements to enhance recognition performance.

The model distinguishes different activities by learning differences in motion dynamics reflected in the sensor signals, such as variations in movement intensity, orientation changes, and temporal consistency. Each input window contains data from a single activity performed by a single user, as recordings were conducted in isolation. This ensures that each segment corresponds to a well-defined activity label, enabling the models to associate characteristic motion patterns with their respective activities. The depth of the networks was selected as a design hyperparameter to balance representation capacity and generalization. Very shallow models (e.g., a single layer) may underfit complex temporal patterns, whereas excessively deep architectures increase computational cost and the risk of overfitting. Therefore, two layers were used for the RNN and LSTM models to provide sufficient hierarchical temporal feature extraction, since recurrent architectures inherently accumulate temporal information through hidden-state propagation across time steps. Increasing the number of recurrent layers beyond this point often yields limited additional benefit while making training less stable [26]. In contrast, Transformer models construct hierarchical representations primarily through stacked self-attention layers rather than recurrence [22]. Consequently, three layers were employed in the Transformer to allow progressive refinement of temporal representations across multiple attention stages.

### 4.1 Recurrent neural network

The DRNN takes as input a 9-dimensional sensor vector at each time step: the accelerometer $\left(a_{x},a_{y},a_{z}\right)$, gyroscope $\left(g_{x},g_{y},g_{z}\right)$, and magnetometer $\left(m_{x},m_{y},m_{z}\right)$ readings. This sequence of vectors forms the input window corresponding to a single activity performed by a single user. The first recurrent layer processes this input sequence and produces a hidden state for each time step. Each hidden state is passed both to the next time step of the same layer and to the second recurrent layer, enabling hierarchical temporal feature extraction. The second layer further refines these features, capturing higher-level temporal patterns that distinguish different activities. The outputs of the second recurrent layer at each time step are passed to the next block of the same layer, allowing the layer to capture temporal dependencies within the sequence (Figure 3). Finally, the outputs of the last blocks from both recurrent layers are concatenated to form an enriched feature vector, which serves as input to the classification layer (details of the classification layer are discussed in Subsection 4.4). This ensures that the classifier receives a representation that combines hierarchical temporal features extracted across both layers, enabling accurate activity prediction.

RNNs capture temporal dependencies by processing input $x_{t}$ and the previous hidden state $h_{t-1}$ to produce a new hidden state $h_{t}$. This hidden state incorporates both current input and past context, allowing RNNs to learn sequential patterns effectively [27, 28]. The recurrence relation for an RNN is (Equation 2):

$$h_{t}=f(W_{xh}x_{t}+W_{hh}h_{t-1}+b_{h})$$ (2)

In a Deep Recurrent Neural Network (DRNN), multiple recurrent layers are stacked, allowing the model to capture more complex temporal dependencies. The recurrence for a two-layer DRNN is defined as (Equations 3 and 4):

$$h_{t}^{\left(1\right)}=f(W_{xh}^{\left(1\right)}x_{t}+W_{hh}^{\left(1\right)}h_{t-1}^{\left(1\right)}+b_{h}^{\left(1\right)})$$ (3)

$$h_{t}^{\left(2\right)}=f(W_{xh}^{\left(2\right)}h_{t}^{\left(1\right)}+W_{hh}^{\left(2\right)}h_{t-1}^{\left(2\right)}+b_{h}^{\left(2\right)})$$ (4)

### 4.2 Long short-term memory (LSTM)

The LSTM-based model follows the same architecture as the DRNN described in the previous section, with the only difference being that the RNN blocks are replaced by LSTM blocks. All other aspects including input dimensions, layer stacking, hidden state flow, and the connection to the classification layer remain the same. Please refer to Section 4.1 for more detailed descrption. LSTM networks improve upon standard RNNs by introducing gating mechanisms that control the flow of information, allowing the model to capture long-term dependencies and mitigate vanishing or exploding gradient issues [29]. Each LSTM block consists of a forget gate, input gate, candidate cell state, cell state, output gate, and hidden state, which together regulate memory at each time step and enable effective encoding and retention of relevant temporal features. The LSTM equations are:

$$f_{t}=\sigma (W_{f}\cdot [h_{t-1},x_{t}]+b_{f})$$ (5)

The **forget gate** ($f_{t}$) determines what portion of the previous cell state ($C_{t-1}$) should be discarded (Equation 5). This gate decides what to ”forget” from the memory.

$$i_{t}=\sigma (W_{i}\cdot [h_{t-1},x_{t}]+b_{i})$$ (6)

The **input gate** ($i_{t}$) controls which new information should be stored in the cell state ($C_{t}$) (Equation 6).

$${\tilde{C}}_{t}=\tanh\;(W_{C}\cdot [h_{t-1},x_{t}]+b_{C})$$ (7)

The **candidate cell state** (${\tilde{C}}_{t}$) proposes potential updates to the cell state based on the current input and previous hidden state (Equation 7).

$$C_{t}=f_{t}*C_{t-1}+i_{t}*{\tilde{C}}_{t}$$ (8)

The **cell state** ($C_{t}$) serves as the long-term memory, updated by combining the previous cell state ($C_{t-1}$) and the new candidate cell state (${\tilde{C}}_{t}$), weighted by the forget and input gates (Equation 8).

$$o_{t}=\sigma (W_{o}\cdot [h_{t-1},x_{t}]+b_{o})$$ (9)

The **output gate** ($o_{t}$) controls what portion of the cell state should be output, determining how much of the memory is passed to the next time step (Equation 9).

$$h_{t}=o_{t}*\tanh\;(C_{t})$$ (10)

The **hidden state** ($h_{t}$) is the output of the LSTM cell at each time step, combining the output gate and the updated cell state to produce the final output (Equation 10).

The LSTM’s gating mechanism helps preserve long-term dependencies, making it especially effective for modeling temporal patterns in Human Activity Recognition.

### 4.3 Transformer

The Transformer architecture [22] revolutionizes sequence modeling by utilizing a self-attention mechanism, allowing the model to capture complex dependencies in input sequences without relying on sequential processing. This is particularly useful for time-series data, where the model can focus on relevant time steps with varying importance. A distinctive feature of the Transformer is the use of *multi-head attention*, where multiple attention heads independently learn different aspects of the sequence in this case different aspect of time series data [22, 28]. The outputs of these heads are then concatenated to form a richer, more comprehensive representation.

The attention mechanism computes a weighted sum of values (V) based on the similarity between queries (Q) and keys (K), where the attention score is given by (Equation 11):

$$\text{Attention}(Q,K,V)=\text{softmax}\left(\frac{QK^{T}}{\sqrt{d_{k}}}\right)V$$ (11)

For multi-head attention, multiple attention operations are performed in parallel and then concatenated, producing the final output (Equation 12):

$$\text{MultiHead}(Q,K,V)=\text{Concat}({\text{head}}_{1},\ldots ,{\text{head}}_{h})W^{O}$$ (12)

**Figure 4:**
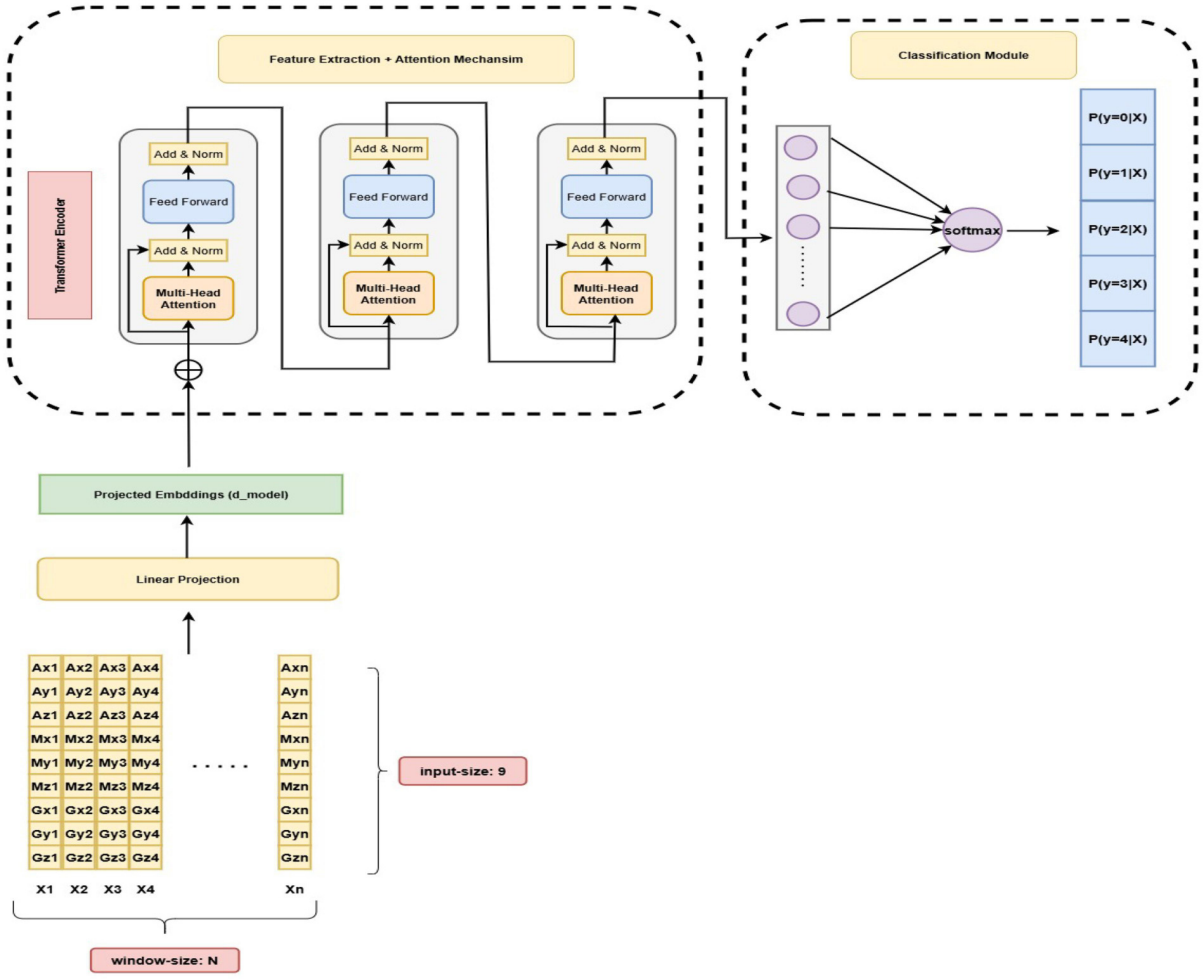
Human activity recognition using transformers.

Figure 4 illustrates the architecture for HAR using Transformers. In our approach, we employ three Transformer encoder blocks, which enable the model to capture complex temporal relationships in sequential activity data. To enhance the input representation, we incorporate a linear projection layer that refines the raw input features by mapping them to a higher-dimensional embedding space. This enhancement enriches the feature representation, allowing the Transformer’s self-attention mechanism to better model the dependencies and variations present in human activity sequences. Unlike dimensionality reduction, this projection focuses on amplifying relevant features, thereby improving the model’s ability to learn subtle activity patterns. Our approach is inspired by the Vision Transformer (ViT) [23], where input data is similarly projected to an embedding space before processing. By leveraging this enhanced input embedding, our architecture is well-suited to effectively recognize and classify human activities with greater accuracy and robustness.

Similar to the DRNN, the model takes as input a sequence of 9-dimensional sensor vectors representing accelerometer, gyroscope, and magnetometer readings for a single activity performed by a user. These raw input features are first mapped into a higher-dimensional embedding space via a linear projection layer, producing enriched representations suitable for the Transformer. Positional encodings are then added to incorporate temporal information into the embeddings. The resulting output is fed into the first Transformer encoder block, and the output of each block serves as the input to the next, allowing the model to progressively capture increasingly abstract temporal dependencies. The final encoder block produces a comprehensive feature vector, which is passed to the classification layer to predict the performed activity.

### 4.4 Classification module

After the input time-series data has passed through the encoder networks, such as RNNs, LSTMs, or Transformers, a feature vector is produced, which encapsulates the learned temporal dependencies and patterns within the data. This feature vector is then passed through a fully connected linear layer, which serves to transform the high-dimensional representation into a more compact form suitable for classification. The linear layer outputs a vector of length five, corresponding to the five activity classes to be predicted. Following this, a softmax activation function is applied, which normalizes the output into a probability distribution. This final output indicates the likelihood of each possible activity occurring based on the given time-series input, enabling the system to accurately perform Human Activity Recognition (HAR). During training, the model is optimized to maximize the probability of the correct (ground truth) activity while minimizing the probabilities assigned to all other classes.

Formally, for a given input X, the model computes the conditional probability of each activity class Y=y as

P(Y=y∣X)=softmax(Wf(X)+b),

where f(X) is the feature vector produced by the encoder, W and b are the weight matrix and bias of the linear layer, and the softmax function normalizes the outputs into a probability distribution over all activity classes. Intuitively, the feature vector f(X), which summarizes the learned temporal patterns from the encoder, is transformed into class scores and then converted into probabilities, allowing the system to predict the most likely activity for the input. The output P(Y=0∣X) specifically represents the probability that the input sequence belongs to the first activity class, with similar interpretations for the other classes.

## 5 Experimental results

### 5.1 Training

The models were implemented using the PyTorch framework, leveraging its flexibility and efficiency for deep learning tasks. Training was conducted on an NVIDIA Tesla P100 GPU to ensure rapid computation and efficient resource utilization. The batch size for all experiments was set to 16, balancing memory usage and model performance. The models were trained under varying experimental configurations without fixing the random seed, which allowed the evaluation to reflect performance across different stochastic training conditions.

For all experiments, the dataset was split into training and test sets, with 80% of the data used for training and 20% reserved for testing. Each model was trained for 50 epochs to ensure sufficient convergence. The Adam optimizer, a robust and adaptive gradient-based optimization algorithm, was used to minimize the loss function. The learning rate was initially set to 0.001 for most training runs; however, for specific RNN configurations, the learning rate was adjusted to improve convergence.

The Cross-Entropy Loss function was employed to evaluate the dissimilarity between the predicted and ground-truth probability distributions. This loss is particularly suitable for multi-class classification tasks, as it ensures the model learns to output probabilities that closely align with the true class labels while penalizing incorrect predictions. For a given input, the loss function is defined as (Equation 13):

$$L=-\sum_{c=1}^{C}y_{c}\log\;({\hat{y}}_{c})$$ (13)

Here, C represents the total number of classes, $y_{c}$ is a binary indicator for the true class c (1 if the class c is the true class, 0 otherwise), and ${\hat{y}}_{c}$ is the predicted probability for class c. By minimizing this loss, the model learns to assign high probabilities to the correct classes, improving its performance on HAR tasks.

The computational cost of training increased with model complexity, which is reflected in the number of learnable parameters summarized in Table 2. The standard RNN required the least computation time due to its simpler recurrent structure and smaller parameter space, followed by the LSTM network, while the Transformer architecture was the most computationally intensive owing to its substantially larger parameter count and attention-based computations. Additionally, experiments using shorter sequence lengths combined with higher overlap ratios resulted in longer training durations, as these settings generated a larger number of training samples. Although Transformer models achieved the best overall classification performance, the improvement relative to LSTMs was modest. This observation indicates diminishing returns in predictive performance as model complexity increases: transitioning from RNN to LSTM yields a notable performance improvement, whereas the shift from LSTM to Transformer provides smaller incremental gains despite significantly higher computational cost.

**Table 2:** Comparison of model complexity.

Model	Number of parameters
RNN	4,709
LSTM	15,173
Transformer	101,381

### 5.2 Results

Tables 3–5 provide detailed experimentation results on various window sizes and overlap ratios across different deep learning models. Specifically, Table 3 presents the results for the Recurrent Neural Network (RNN), Table 4 illustrates the performance of Long Short-Term Memory (LSTM) networks, and Table 5 showcases the effectiveness of the Transformer model in stress-oriented Human Activity Recognition (HAR).

**Table 3:** Performance metrics for different window sizes and overlap ratios for recurrent neural network.

Window size	Overlap	Accuracy	Precision	Recall	F1-Score
16	50%	0.891	0.8907	0.891	0.8907
	60%	0.8948	0.8954	0.8948	0.8949
	70%	**0.9109**	**0.9115**	**0.9109**	**0.911**
	80%	0.8999	0.9004	0.8999	0.9001
32	50%	0.7789	0.7802	0.7789	0.7794
	60%	0.8795	0.88	0.8795	0.8793
	70%	0.8762	0.8776	0.8762	0.876
	80%	**0.924**	**0.9246**	**0.924**	**0.924**
64	50%	0.5991	0.5917	0.5991	0.5852
	60%	**0.8676**	**0.8687**	**0.8676**	**0.8678**
	70%	0.7393	0.7629	0.7393	0.7279
	80%	0.8047	0.8263	0.8047	0.8034
128	50%	0.6008	0.592	0.6008	0.5523
	60%	0.5378	0.5617	0.5378	0.4959
	70%	**0.6878**	**0.7022**	**0.6878**	**0.6697**
	80%	0.6506	0.6476	0.6506	0.635
256	50%	0.4632	0.4206	0.4632	0.4146
	60%	0.5802	0.5745	0.5802	0.5504
	70%	0.6175	0.6323	0.6175	0.5872
	80%	**0.667**	**0.6641**	**0.667**	**0.6413**
512	50%	0.523	0.5301	0.523	0.4677
	60%	0.6186	0.6207	0.6186	0.6146
	70%	0.629	0.6305	0.629	0.6133
	80%	**0.7225**	**0.7233**	**0.7225**	**0.718**

The bold values represent the best-performing metrics for the respective models and experimental settings.

**Table 4:** Performance metrics for different window sizes and overlap ratios for LSTM.

Window size	Overlap	Accuracy	Precision	Recall	F1-Score
16	50%	0.9409	0.9411	0.9409	0.9409
	60%	0.9415	0.9417	0.9415	0.9415
	70%	0.9490	0.9490	0.9490	0.9489
	80%	**0.9610**	**0.9617**	**0.9610**	**0.9610**
32	50%	0.9409	0.9417	0.9409	0.9408
	60%	0.9419	0.9420	0.9419	0.9419
	70%	0.9622	0.9623	0.9622	0.9622
	80%	**0.9758**	**0.9759**	**0.9758**	**0.9758**
64	50%	0.9222	0.9226	0.9222	0.9220
	60%	0.9479	0.9481	0.9479	0.9479
	70%	0.9471	0.9475	0.9471	0.9472
	80%	**0.9736**	**0.9738**	**0.9736**	**0.9735**
128	50%	0.8581	0.8588	0.8581	0.8579
	60%	0.9093	0.9109	0.9093	0.9096
	70%	0.9513	0.9515	0.9513	0.9512
	80%	**0.9778**	**0.9784**	**0.9778**	**0.9778**
256	50%	0.7820	0.7830	0.7820	0.7796
	60%	0.8418	0.8410	0.8418	0.8408
	70%	0.8775	0.8780	0.8775	0.8758
	80%	**0.9303**	**0.9306**	**0.9303**	**0.9302**
512	50%	0.7644	0.7636	0.7644	0.7631
	60%	0.7814	0.7795	0.7814	0.7750
	70%	0.7809	0.7854	0.7809	0.7781
	80%	**0.8713**	**0.8715**	**0.8713**	**0.8713**

The bold values represent the best-performing metrics for the respective models and experimental settings.

**Table 5:** Performance metrics for different window sizes and overlap ratios for transformer.

Window size	Overlap	Accuracy	Precision	Recall	F1-Score
16	50%	0.9428	0.9428	0.9428	0.9428
	60%	0.9521	0.9525	0.9521	0.9521
	70%	0.9564	0.9570	0.9564	0.9562
	80%	**0.9656**	**0.9658**	**0.9656**	**0.9656**
32	50%	0.9487	0.9490	0.9487	0.9486
	60%	0.9490	0.9493	0.9490	0.9490
	70%	0.9585	0.9591	0.9585	0.9585
	80%	**0.9772**	**0.9772**	**0.9772**	**0.9772**
64	50%	0.9346	0.9348	0.9346	0.9346
	60%	0.9606	0.9608	0.9606	0.9606
	70%	0.9602	0.9604	0.9602	0.9601
	80%	**0.9731**	**0.9737**	**0.9731**	**0.9730**
128	50%	0.9324	0.9336	0.9324	0.9320
	60%	0.9600	0.9602	0.9600	0.9599
	70%	0.9619	0.9625	0.9619	0.9619
	80%	**0.9783**	**0.9784**	**0.9783**	**0.9783**
256	50%	0.9183	0.9246	0.9183	0.9186
	60%	0.9385	0.9397	0.9385	0.9376
	70%	**0.9669**	**0.9689**	**0.9669**	**0.9670**
	80%	0.9640	0.9653	0.9640	0.9639
512	50%	0.9598	0.9602	0.9598	0.9597
	60%	0.9721	0.9735	0.9721	0.9722
	70%	0.9717	0.9719	0.9717	0.9717
	80%	**0.9761**	**0.9774**	**0.9761**	**0.9761**

The bold values represent the best-performing metrics for the respective models and experimental settings.

Each table presents a detailed evaluation of model performance across six distinct window sizes: 16, 32, 64, 128, 256, and 512, covering a timespan ranging from 0.02 seconds to 10 seconds. To comprehensively assess the effect of window overlaps, four separate experiments were conducted for each window size using overlap ratios of 50%, 60%, 70%, and 80%. The performance of each experimental setup is measured using four key evaluation metrics Accuracy, Precision, Recall, and F1-score which collectively provide a holistic view of the model’s classification effectiveness.

#### 5.2.1 Impact of window size on model performance

**Figure 5:**
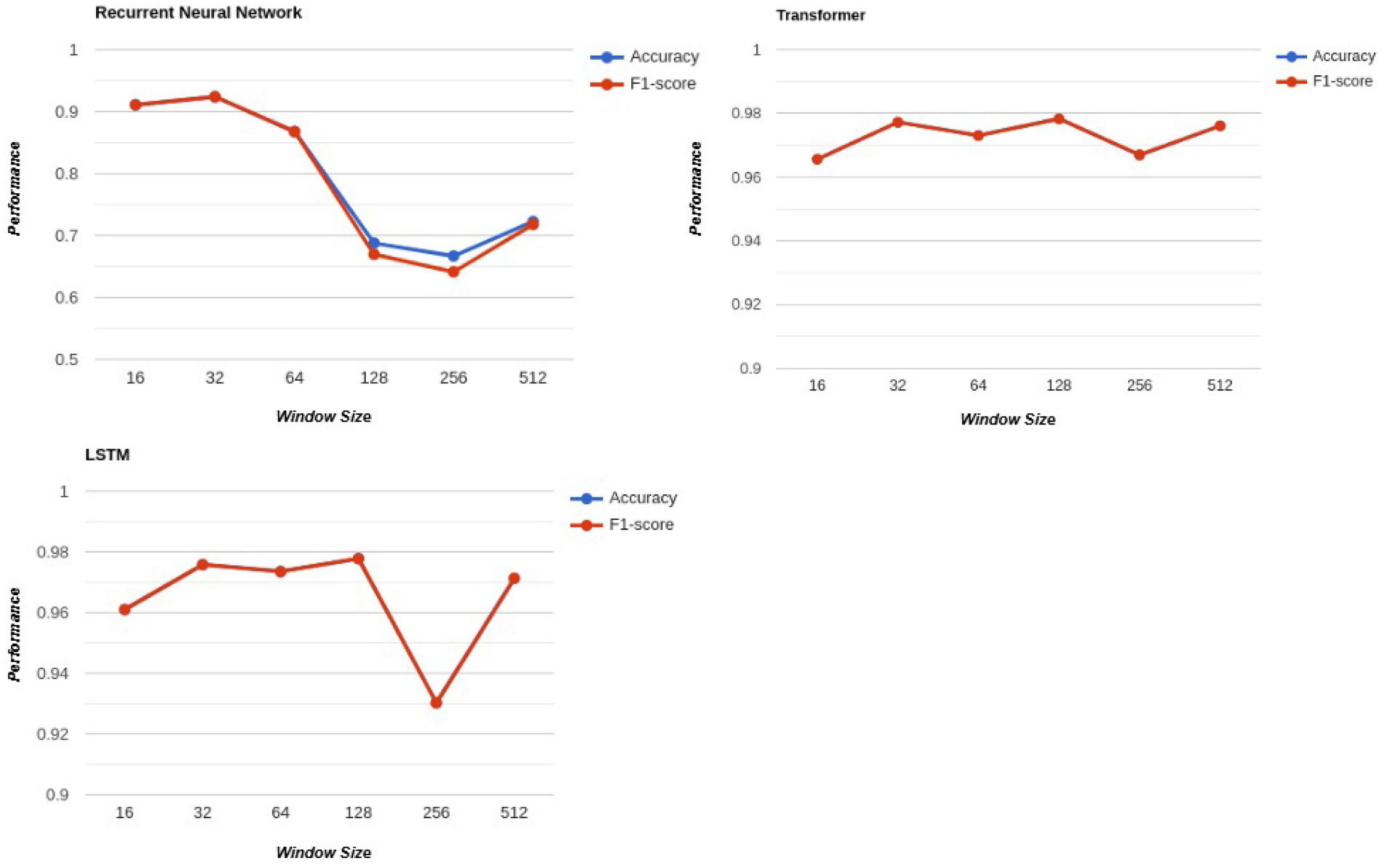
Effect of window size on classification accuracy for different deep learning models.

Figure 5 provides a comprehensive analysis of the effect of window size on the performance of various deep learning architectures, including Recurrent Neural Networks (RNNs), Long Short-Term Memory networks (LSTMs), and Transformers.

The impact of window size on model performance reveals key insights into how each architecture handles temporal dependencies. For RNNs, smaller window sizes led to better performance as compared to the larger window sizes. The architecture excelled at capturing patterns and dependencies in short sequences, with peak performance observed at a window size of 32. However, as the window size increased, the performance of RNNs declined significantly. This decline can be attributed to the fundamental limitation of RNNs in managing long-term dependencies. RNNs process sequences sequentially, and as the length of the sequence grows, the gradients that carry temporal information diminish, leading to the well-known vanishing gradient problem. Consequently, RNNs struggle to retain meaningful information from earlier parts of long sequences, which hampers their ability to effectively process large window sizes.

LSTMs, designed to address the limitations of RNNs, showed a clear trend of improving performance as window size increased, peaking at a window size of 128 due to their ability to store and process longer temporal dependencies using gating mechanisms. Beyond this point, their performance declined, likely due to the increased complexity of processing overly long sequences, which can overwhelm the gating mechanisms and hinder the model’s ability to differentiate critical temporal patterns. However, an unexpected performance increase at a window size of 512 suggests that longer sequences might occasionally reveal overarching trends or periodic patterns that shorter sequences cannot capture. This anomaly highlights the nuanced behavior of LSTMs, where their performance is influenced not only by their architecture but also by the characteristics of the dataset. While LSTMs generally perform best at intermediate window sizes, the results underline the importance of empirical testing to balance performance and computational efficiency and to explore the potential benefits of larger window sizes when applicable. Recent studies in Human Activity Recognition have demonstrated that combining convolutional layers with recurrent architectures can improve feature learning by capturing local temporal dependencies before sequential modeling. Architectures such as DeepConvLSTM [27] employ convolutional layers to reduce the input dimensionality and extract discriminative local features prior to LSTM processing. Such projection mechanisms can help mitigate performance degradation when larger temporal windows are used.

Transformers demonstrated exceptional performance across all window sizes, with their optimal results achieved at a window size of 128. Leveraging their self-attention mechanism, they can process sequences in parallel and capture both short-term and long-term dependencies. However, their performance did not follow a strictly linear pattern. Instead, it exhibited minor fluctuations, with accuracy values ranging between 0.969 and 0.979, reflecting small increases and decreases as the window size varied. These fluctuations, while noticeable, were relatively minor, suggesting that Transformers remained highly stable and effective across all window sizes. The observed pattern indicates the model’s ability to adapt to different temporal spans, with the best performance achieved at window size 128, demonstrating its versatility and robustness in handling long-term dependencies.

#### 5.2.2 Effect of overlap ratio on performance metrics

**Figure 6:**
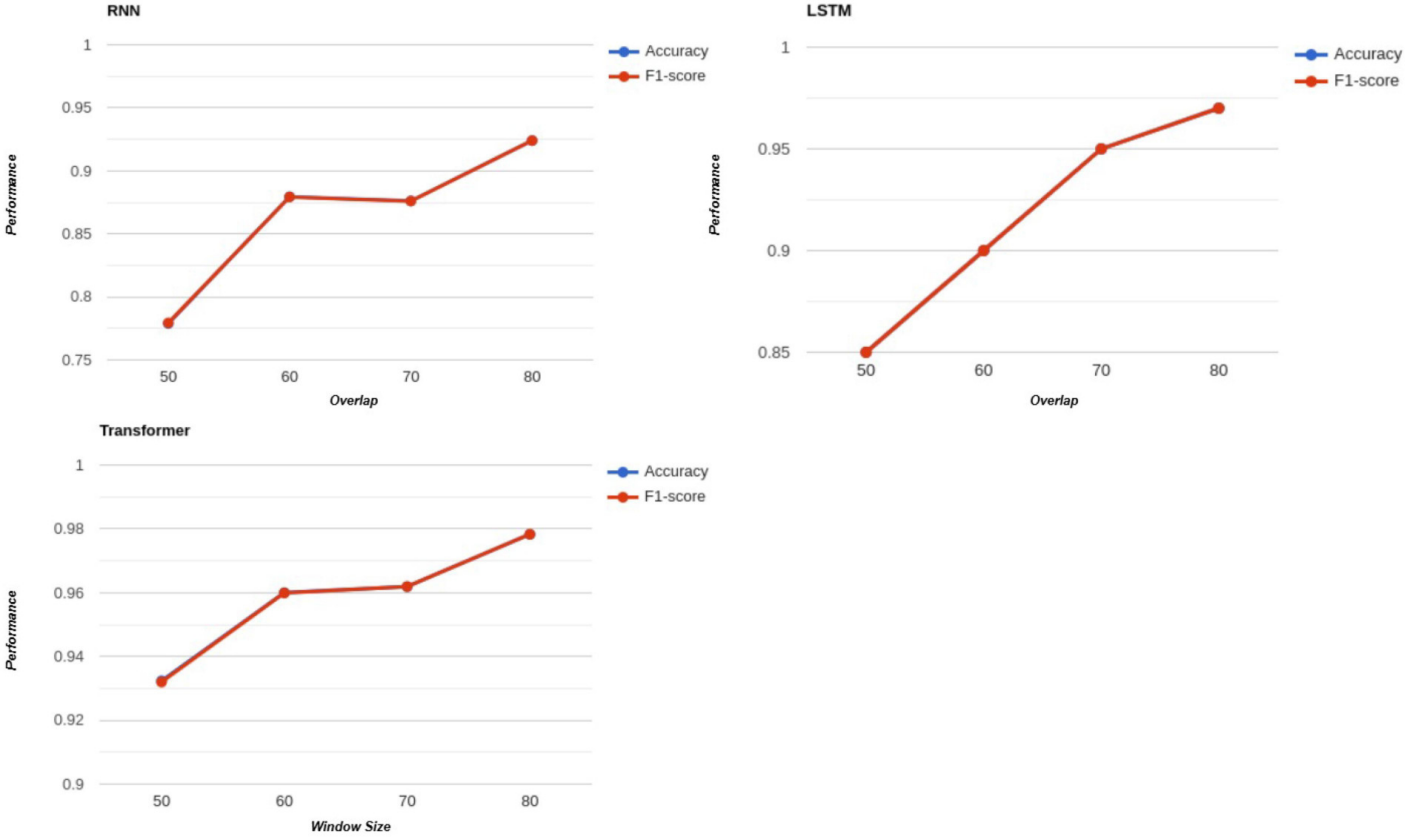
Effect of overlap ratio on classification accuracy for different deep learning models.

We conducted experiments to evaluate the performance of each deep learning model RNNs, LSTMs, and Transformers across four distinct overlap ratios: 50%, 60%, 70%, and 80%. This analysis was performed for all window sizes, ensuring that the influence of overlap on model performance could be thoroughly investigated. Figure 6 visualizes how these overlap ratios impact performance. RNNs performed best with a window size of 32, while both LSTMs and Transformers achieved their highest performance with a window size of 128.

From the figure, we can observe a general trend: increasing the overlap ratio tends to enhance the performance of all three models. This improvement is most evident in higher overlap scenarios, where RNNs, LSTMs, and Transformers consistently demonstrate better results. However, it is important to note that this trend is not perfectly uniform across all cases. Variations can be observed, as detailed in Tables 3–5. These minor inconsistencies highlight that while the overlap ratio is an influential factor, its effect may depend on other elements, such as the model architecture and the characteristics of the dataset.

The observed improvements with higher overlap ratios can be attributed to several factors. One key reason is that increasing the overlap ratio effectively generates more training examples from the same dataset. By having overlapping segments, the models are exposed to similar patterns and temporal dependencies more frequently, allowing them to learn and generalize better. Additionally, higher overlap reduces the risk of missing critical transitions or features that may lie near the boundaries of non-overlapping segments. This is particularly important in temporal data, where subtle patterns might be critical for accurate predictions.

However, it is also important to recognize that increasing the overlap ratio comes with trade-offs. While more training examples are beneficial, excessive overlap can introduce redundancy, where the model repeatedly sees nearly identical examples. This may lead to diminishing returns in performance improvements and an increase in computational costs. Therefore, determining an optimal overlap ratio is essential to balance the benefits of improved performance and the potential drawbacks of computational overhead.

#### 5.2.3 Performance comparison across deep learning architectures & previous work

**Figure 7:**
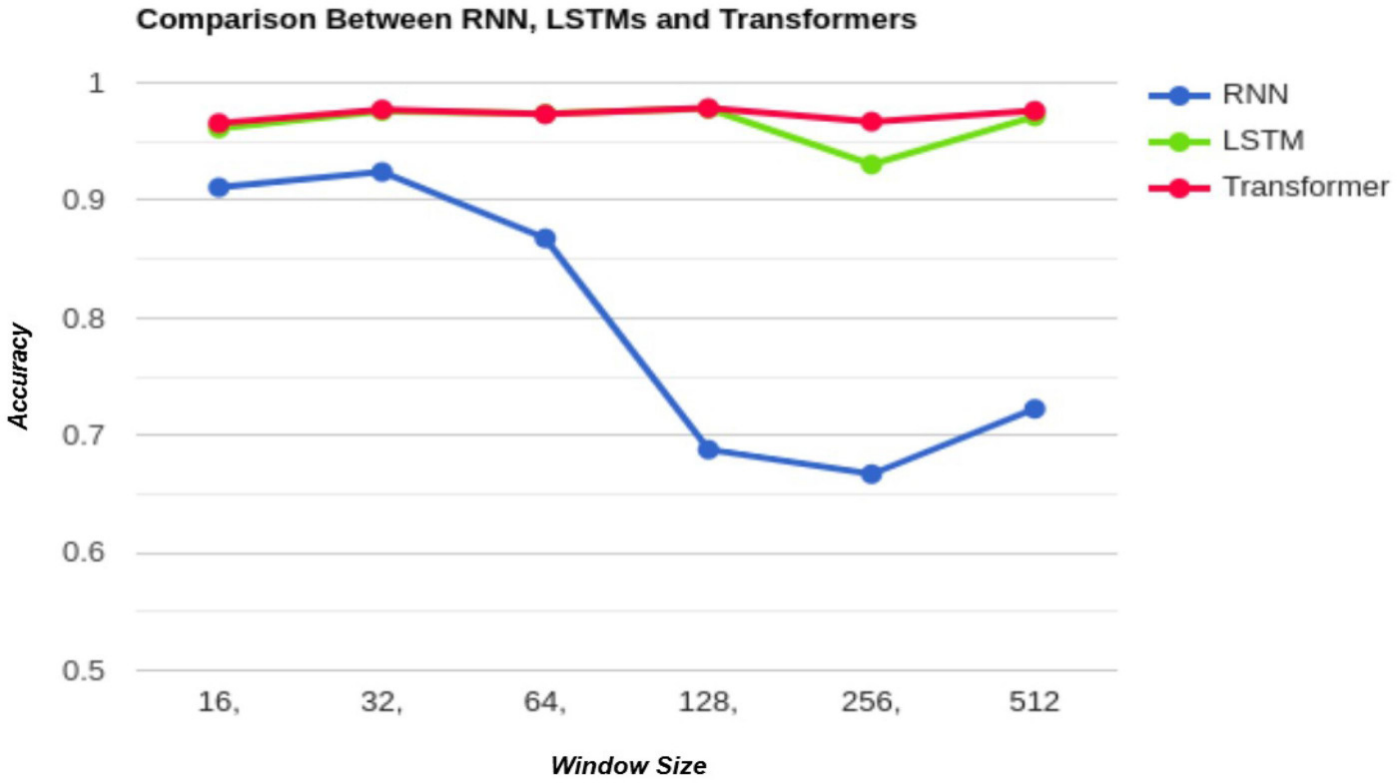
Comparison of classification accuracy among RNN, LSTM, and Transformer models across different experimental settings.

**Figure 8:**
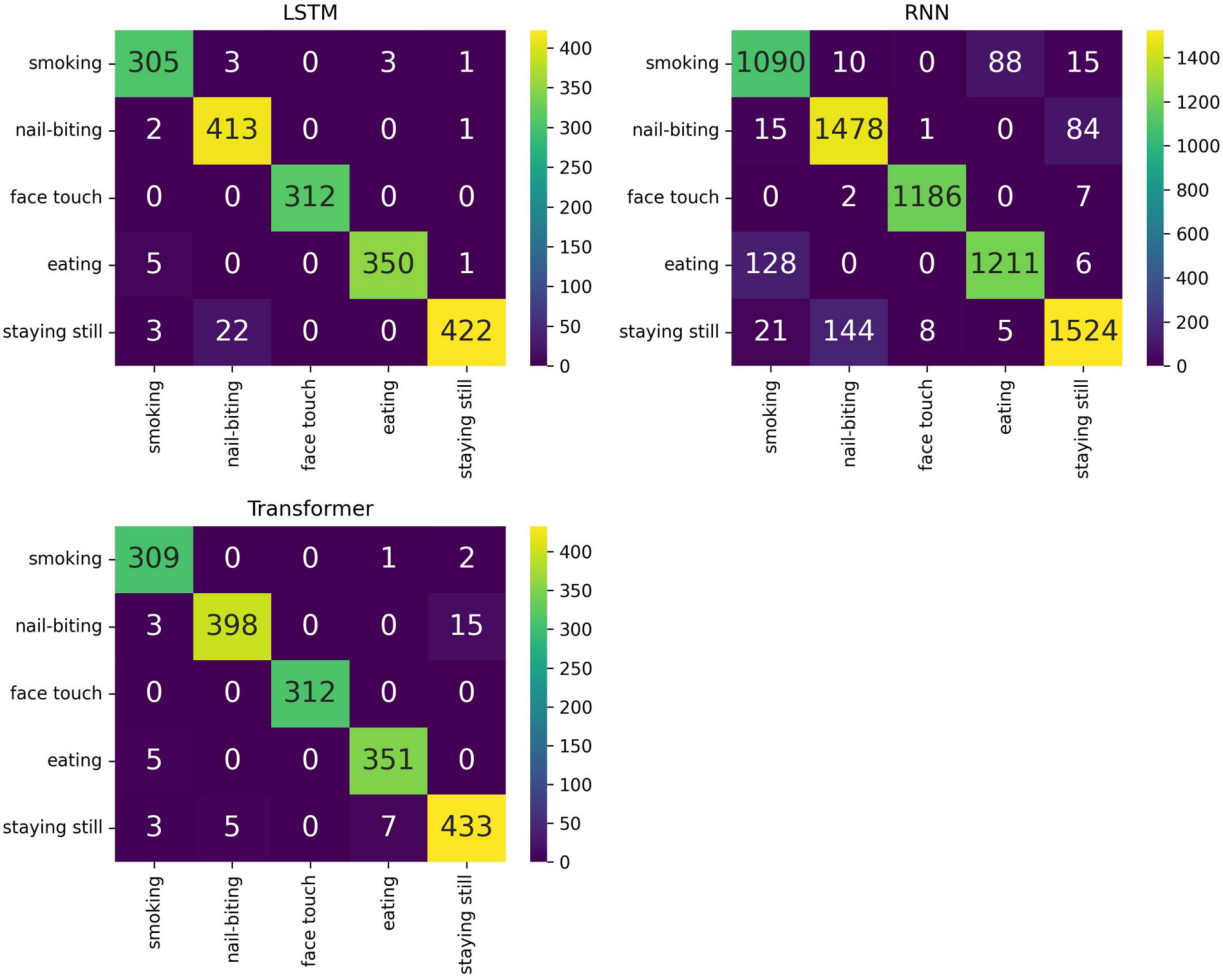
Confusion matrices on the test dataset for the best-performing models: LSTM, RNN, and transformer.

Figure 7 presents a graphical comparison of the performance of RNN, LSTM, and Transformer models based on accuracy. Figure 8 depicts the confusion matrices corresponding to the afore mentioned models. The Transformer consistently outperforms the other architectures across all window sizes while requiring greater computational resources, as outlined in Section 5.1. LSTMs follow closely behind, showing similar performance to the Transformer and securing the second position in terms of efficiency. In contrast, Recurrent Neural Networks (RNNs) yield the lowest accuracy, making them the least effective architecture in this comparison.

In our previous study [4] we utilized the dataset [9] and explores various experiments with different window sizes. We evaluated several architectures of Deep Neural Networks (DNN) alongside conventional Machine Learning algorithms. The best performance in that work was achieved using a Deep Neural Network (DNN), which resulted in an accuracy of 93.5% on the test dataset. In contrast, the present work achieved a superior result with Transformers, reaching an accuracy of 97.61%. This represents an improvement of approximately 4.1% over the previous work.

Table 6 highlights the performance of our methodology compared to previous efforts in this domain. The results clearly show that LSTMs and Transformers achieved higher accuracy than earlier methods, which emphasizes their effectiveness. These findings demonstrate the potential of these advanced models to address the challenges in this task, delivering improved outcomes and showcasing the impact of our approach.

**Table 6:** Comparison of model performance for stress activity recognition.

Source	Model	Accuracy
Prior study [4]	Deep neural network (DNN)	93.5%
Current work	RNN	92.4%
Current work	**LSTM**	**97.78%**
Current work	**Transformer**	**97.83%**

The bold values represent the best-performing metrics for the respective models and experimental settings.

## 6 Conclusion

In this study, we explored the use of sensor-based time-series data for HAR, with a focus on detecting stress-related activities such as face-touching, eating, and nail-biting. Through the application of advanced deep learning architectures, including Recurrent Neural Networks (RNNs), Long Short-Term Memory (LSTM) networks, and Transformers, we demonstrated their effectiveness in identifying subtle behavioral patterns associated with stress. Our extensive experimentation highlighted the significance of factors like window size and overlap, which directly influenced the classification accuracy of these models. Notably, our findings showed that Transformers outperformed other architectures, achieving a substantial improvement in accuracy, surpassing previous work by nearly 4.1%. This work emphasizes the potential of leveraging wearable sensor data combined with deep learning to develop efficient systems for real-time stress detection, which could be valuable for enhancing mental health monitoring and supporting personalized well-being interventions. The primary limitation of this study is the absence of non–stress-related diurnal activities in the dataset. Incorporating such activities and interleaving them with stress-related tasks would enhance the robustness, generalizability, and reliability of the proposed model. Additionally, the use of overlapping windows in time-series datasets may introduce a potential risk of data leakage if not carefully managed. Future work will therefore focus on integrating a broader range of daily-life activities and adopting Leave-One-Subject-Out (LOSO) cross-validation to better evaluate subject-independent performance. These improvements will also support the development of a more ecologically valid and real-time deployable system.

## Data Availability

Publicly available datasets were analyzed in this study. This data can be found here: https://data.mendeley.com/datasets/2dn3hpbm5m/1
